# Supplementary material for: Collaborative intelligence in AI: Evaluating the performance of a council of AIs on the USMLE
Source: PLOS Digit Health. 2025 Oct 9;4(10):e0000787. doi: 10.1371/journal.pdig.0000787 (PMC12510544; doi:10.1371/journal.pdig.0000787)
Supplement: S2 File — (ZIP) [file pdig.0000787.s002.zip › S2 Data Raw data files containing unprocessed question inputs and LLM outputs including transcripts of deliberations/STEP3_Question-57-1_Type-MC-NJ.docx]

**Facilitator** **[Round 1]:** A 45-year-old man comes to the office for a follow-up examination 2 days after he was seen in the emergency department for a generalized tonic-clonic seizure. He was prescribed chlordiazepoxide upon being discharged but did not fill the prescription. The patient says that this was his third seizure in the past 2 years; prior to that he had no history of seizures. Medical history is also remarkable for a 10-year history of daily ingestion of two six-packs of beer. He has not had any alcoholic beverages for the past 5 days. He takes no medications. The patient says that his seizures usually occur after he abstains from alcohol for a period of time. Vital signs are normal. Physical examination shows scattered spider angiomata on his chest and abdomen. Liver is enlarged, smooth, and nontender. CT scan of the head with contrast done in the emergency department 2 days ago showed no abnormalities. Which of the following is the most appropriate next step? (A) Lumbar puncture for examination of cerebrospinal fluid (B) MRI of the brain (C) Repeat CT scan of the head in 1 week (D) Sleep-deprived electroencephalography (E) No further evaluation is necessary

**AI_A:** RESTATING THE QUESTION: We have a 45-year-old male patient who had a recent generalized tonic-clonic seizure, the third of such episodes in the past two years. In addition, he has a significant history of alcohol consumption, now being 5 days sober. He was prescribed chlordiazepoxide after the most recent seizure but did not take it. The patient reports that his seizures occur when he stops drinking. His vitals are normal, though physical examination reveals spider angiomata on his chest and abdomen along with an enlarged, smooth, and nontender liver. A recent CT scan of his head showed no abnormalities. The question is, what is the next best step in his management? PLANNING: The fact that the patient's seizures seem to be linked with periods of alcohol abstinence suggests a diagnosis of alcohol withdrawal seizures. In addition, the patient has signs of possible chronic liver disease (spider angiomata, enlarged liver), a common consequence of prolonged heavy alcohol use. Chlordiazepoxide, a benzodiazepine, is commonly used for managing symptoms of alcohol withdrawal such as seizures, suggesting that this was the initial diagnosis made in the emergency department. The challenge is determining what further investigations, if any, are needed at this point. CLINICAL REASONING: Alcohol withdrawal seizures typically occur within 6-48 hours following cessation or reduction of heavy alcohol use; the timing of the patient's seizures fits within this window. The history, combined with the physical findings, support a diagnosis of alcohol withdrawal seizures and potential alcoholic liver disease. An already performed CT scan found no other cause for the seizures, making something like a brain tumor or stroke less likely. Additional imaging or invasive procedures, such as an MRI or lumbar puncture, do not seem clinically other than in the absence of other findings. CONCLUDING: The mainstay of treatment in this situation seems like it should be managing the patient's alcohol withdrawal, which includes managing his seizures, as well as addressing his chronic alcohol use. Given that the CT scan did not reveal any intracranial abnormalities, it's likely that these seizures are due to alcohol withdrawal, and no further diagnostic intervention would be necessary at this point, making choice (E) the most plausible answer. RESPONSE: Therefore, the most appropriate next step in this patient's care seems to be (E) no further evaluation is necessary, as it seems appropriate to treat him for alcohol withdrawal seizures and start addiction therapy for his chronic alcohol use.

**AI_C:** RESTATING THE QUESTION: We're dealing with a 45-year-old male patient who had a generalized tonic-clonic seizure recently and had 2 more similar episodes in the past 2 years. Notably, he has a history of chronic heavy alcohol use, but has been abstinent for the past 5 days. The patient stated his seizures usually occur when he stops drinking alcohol. On examination, he has signs of chronic liver disease such as spider angiomata and an enlarged liver. A recent head CT scan showed no abnormalities. The question is asking for the most suitable next evaluation step. PLANNING: Several key points can be drawn from the provided information. First, the frequent heavy intake of alcohol and sudden abstinence is crucial in understanding the condition. Alcohol withdrawal can lead to seizures, typically within 6-48 hours after the last drink. Second, the presence of spider angiomata and an enlarged, smooth liver points to cirrhosis, a likely end-result of chronic alcohol misuse. However, the lack of head abnormalities in the recent CT scan published leaves little to guess regarding a CNS cause of the seizures. The challenge here is to define which approach would most clinically benefit the patient. CLINICAL REASONING: The patient's seizures appear to be highly linked to periods of alcohol abstinence, suggesting that they may be alcohol withdrawal seizures. These seizures are part of the alcohol withdrawal syndrome, which can occur upon the abrupt discontinuation of alcohol in a patient who has been drinking heavily for a long time. The normal CT head scan also supports this as it rules out intracranial structural causes of seizures such as tumors or bleeds. Given his history and the absence of neurological findings, optimally managing his withdrawal symptoms should be considered a priority. CONCLUDING: Further investigations such as a lumbar puncture, brain MRI, or repeat CT scan are likely to add little to the primary care of this patient. However, the patient would immensely benefit from an evaluation of his alcohol withdrawal symptoms and management of the same as necessary - possibly starting on a alcohol withdrawal regimen. It is in his best interest as well since the seizures seem mainly due to periods of alcohol abstinence. RESPONSE: Therefore, the answer is (E) No further evaluation is necessary. The patient's symptoms can be addressed by managing his alcohol withdrawal. This includes monitoring withdrawal symptoms, starting medical therapy as indicated (possibly with a benzodiazepine which was originally prescribed to him), and referring him for alcohol counselling and support.

**AI_B:** RESTATING THE QUESTION: A 45-year-old man, with a seizure episode and history of alcoholism, presents in the office. Despite being prescribed chlordiazepoxide, which is a medication used in the treatment of withdrawal symptoms in alcoholism, he did not take his medication. He reports that the seizures usually occur when he stops drinking. He has stopped consuming alcohol for the past 5 days. Physical examination reveals signs of chronic liver disease, in this case, spider angiomata, and an enlarged and smooth liver. A recent CT scan didn't show any abnormalities in his head. We need to choose the most appropriate next step in his management. PLANNING: First, we assume that the patient's seizures are due to his history of alcoholism, given the timing of his seizures and his refusal to take his prescribed medication. However, we should also assess other potential causes, including neurological disorders that might be detected by further imaging (MRI), infections prompting a lumbar puncture for CSF analysis, or epileptic disorders that could be revealed through EEG. Considering all these, the patient's chronic alcohol ingestion, withdrawal symptoms, and the recent CT scan that found no abnormalities, alcohol withdrawal seems the most likely cause and warrants clinical prioritization over further neurologic evaluations. CLINICAL REASONING: The onset of this patient's seizures in the context of recent alcohol cessation after a long history of heavy usage points strongly toward alcohol withdrawal seizures. These seizures generally occur within 6-48 hours after cessation of alcohol and are a constituent of the alcohol withdrawal syndrome, which may also include nausea, hallucinations, agitation, and even delirium tremens. More importantly, the CT scan, a vital part in the evaluation of a new onset seizure, has already been performed and was normal, further establishing alcohol as the likely etiology. CONCLUDING: Given the timing of the seizures in context to alcohol cessation, his history of chronic alcohol usage, and the unremarkable CT scan, it appears that alcohol withdrawal is the likely cause of his seizures. Further investigations like EEG, MRI, repeat CT, or lumbar puncture may not add significant value unless there is suspicion of other neurological pathologies. However, withholding alcohol in this case could result in severe withdrawal, including delirium tremens, and should always be managed appropriately. RESPONSE: Therefore, the most appropriate next step would not be further investigation but to start the patient on the prescribed chlordiazepoxide (option E: No further evaluation is necessary), which would help manage his alcohol withdrawal symptoms, potentially prevent further seizures, and reduce the risk of progression to more dangerous withdrawal syndromes. It is also important to remember that these patients would need comprehensive treatment for alcohol dependence, including psychosocial interventions.

**AI_D:** RESTATING THE QUESTION: Our patient is a 45-year-old man with a history of chronic alcohol abuse and recent generalized tonic-clonic seizures. He has experienced three seizures in the past two years, all occurring after periods of alcohol abstinence. His physical examination reveals spider angiomata and an enlarged liver, suggestive of chronic liver disease, presumably alcohol-related. A recent head CT scan revealed no abnormalities. The patient was prescribed chlordiazepoxide, typically used to manage alcohol withdrawal, but he did not take it. We are asked to determine the most appropriate next step in his management. PLANNING: The main assumption is that his seizures are due to alcohol withdrawal, which is common in individuals with a history of chronic alcohol misuse, especially after periods of abstinence. One alternative assumption could be that the seizures have another etiology, such as an underlying neurological condition, requiring further imaging studies like MRI (option B) or repeat CT scan (option C), analysis of cerebrospinal fluid via lumbar puncture (option A), or sleep-deprived electroencephalography (option D). However, given his history and the absence of any other neurological symptoms, it's more likely that his seizures are due to alcohol withdrawal. CLINICAL REASONING: These seizures occur because alcohol affects the central nervous system by enhancing the inhibitory effect of gamma-aminobutyric acid (GABA) and suppressing the excitatory effect of glutamate. Abrupt cessation or reduction in alcohol intake leads to a rebound increase in neuronal excitability due to decreased GABAergic and increased glutamatergic transmission, potentially leading to seizures. Notably, the patient's history of chronic alcohol miss use, along with his correlating seizure incidents with alcohol abstinence, strengthens the clinical impression that these seizures are alcohol withdrawal-related. In such cases, the importance of managing withdrawal symptoms and preventing further seizures with medications such as chlordiazepoxide cannot be overstated. CONCLUDING: Based upon the clinical presentation, the history of chronic alcohol misuse, and the lack of neurological abnormalities seen on the CT scan, the seizure is most likely due to alcohol withdrawal. Further evaluation, including imaging, lumbar puncture, or electroencephalography, might not be necessary as it wouldn't change the management plan, which should include managing withdrawal symptoms and preventing further seizures with appropriate medications. RESPONSE: (E) No further evaluation is necessary. Instead, the attention should be focused on managing his withdrawal symptoms and preventing further seizures, which would involve the usage of prescribed medications such as chlordiazepoxide that the patient was initially given but did not take. The patient's alcohol abuse should also ideally be addressed with proper counseling and support services to aid in alcohol cessation.

**AI_E:** RESTATING THE QUESTION: A 45-year-old male patient visited our office for a follow-up check-up. Recently, he experienced a generalized tonic-clonic seizure, an event characterized as a type of seizure that affects the entirety of the brain. Despite being given a chlordiazepoxide prescription upon discharge, he never filled the prescription. So far, in the last 2 years, he's reported three such seizures, but prior to this time frame, no history of seizures exists. The patient's medical history also sheds light on a chronic alcohol ingestion behavior that extends back a decade. However, he has been completely alcohol-free for the past 5 days and is currently not on any particular medication. According to the patient's feedback, his seizure attacks correlate with his periods of alcohol abstinence. Upon medical examination, his vital signs seem normal, yet there is the presence of spider angiomata scattered around his chest and abdomen. This is a direct effect of changes in the liver caused by alcohol-induced liver disease, characterized by visible red spots that resemble a spider's web. The liver itself is enlarged, smooth, and nontender, indicative of potentially chronic liver disease. Recent CT scans, however, fail to show any noticeable abnormalities. Given the aforementioned circumstances, we need to identify the best possible next step for the patient. PLANNING: The default assumption would be that the seizures are directly linked to his alcohol ingestion history. In this context, alcohol withdrawal syndrome is a plausible cause. When making this assumption, it's necessary to remember that abrupt cessation of alcohol can lead to withdrawal seizures generally within 6-48 hours post-abstinence. Characterizing the mechanism of alcohol withdrawal syndrome as a result of unopposed excitatory neurotransmission may help set the clinical priority based on the patient's symptoms. An alternate assumption could point to another underlying neurological disease that's causing seizures. However, given the absence of traditionally associated symptoms and the results of the recent CT scan, this is less likely. CLINICAL REASONING: With the alcohol withdrawal seizures theory in mind, let's assess the patient's symptoms and behaviors. Our patient seems to fit the symptomatology and timeline for alcohol withdrawal seizures perfectly as his seizures occur when he abstains from alcohol, aligning with the typical 6-48 hour timeframe for withdrawal seizures. Further, the relevant medical history of chronic alcohol ingestion and the physical examination findings, which suggest chronic liver disease (spider angiomata, enlarged smooth liver), support chronic alcohol abuse. Additionally, it's important to note that chlordiazepoxide, a benzodiazepine, was prescribed but not filled by the patient. Benzodiazepines are the treatment of choice for withdrawal seizures as they reduce the likelihood of further seizures and can prevent the progression to delirium tremens, a severe form of alcohol withdrawal that can be fatal. With all this information combined, we can hypothesize that our patient's seizures are most likely due to alcohol withdrawal. CONCLUDING: Thus, it seems reasonable to conclude that this patient's seizures are likely withdrawal seizures due to chronic alcohol abuse. Considering that the CT scan failed to reveal any abnormalities and the patient fits the textbook presentation for withdrawal seizures, there seems to be no immediate need for further brain imaging or invasive procedures, like lumbar puncture, at this point. RESPONSE: Therefore, the most appropriate next step would be for the patient to start his prescribed medication, chlordiazepoxide (Option E - No further evaluation is necessary). It would also be wise to recommend referral to addiction treatment services and counseling to aid in safely managing withdrawal symptoms and promoting long-term recovery.

**Facilitator** The consensus among the AI responses is that the most appropriate next step in the management of this 45-year-old male patient, who has a history of chronic alcohol consumption and recent generalized tonic-clonic seizures, is option (E) No further evaluation is necessary. The patient's seizures appear to be linked to periods of alcohol abstinence, suggesting a diagnosis of alcohol withdrawal seizures. This is further supported by the patient's history of chronic alcohol use and the physical examination findings suggestive of chronic liver disease. The recent CT scan of his head showed no abnormalities, making other causes for the seizures, such as a brain tumor or stroke, less likely. Therefore, the focus should be on managing the patient's alcohol withdrawal symptoms, which includes managing his seizures with the prescribed chlordiazepoxide, and addressing his chronic alcohol use through addiction therapy and support. Further investigations such as a lumbar puncture, brain MRI, or repeat CT scan are likely to add little to the primary care of this patient.
